# Development of Prediction Models for New Integrated Models and a Bioscore System to Identify Bacterial Infections in Systemic Lupus Erythematosus

**DOI:** 10.3389/fcimb.2021.620372

**Published:** 2021-03-01

**Authors:** Xvwen Zhai, Min Feng, Hui Guo, Zhaojun Liang, Yanlin Wang, Yan Qin, Yanyao Wu, Xiangcong Zhao, Chong Gao, Jing Luo

**Affiliations:** ^1^Clinical Skills Teaching Simulation Hospital, Shanxi Medical University, Jinzhong, China; ^2^Department of Rheumatology, The Second Hospital of Shanxi Medical University, Taiyuan, China; ^3^Division of Nephrology, Department of Medicine, The Second Hospital of Shanxi Medical University, Taiyuan, China; ^4^Division of Nephrology, Department of Medicine, The Shenzhen Baoan Shiyan People’s Hospital, Shenzhen, China; ^5^Department of Pathology, Brigham and Women’s Hospital and Harvard Medical School, Boston, MA, United States

**Keywords:** systemic lupus erythematosus, bacterial infection, lupus flare, receiver operating characteristic, bioscore

## Abstract

**Objectives:**

Distinguishing flares from bacterial infections in systemic lupus erythematosus (SLE) patients remains a challenge. This study aimed to build a model, using multiple blood cells and plasma indicators, to improve the identification of bacterial infections in SLE.

**Design:**

Building PLS-DA/OPLS-DA models and a bioscore system to distinguish bacterial infections from lupus flares in SLE.

**Setting:**

Department of Rheumatology of the Second Hospital of Shanxi Medical University.

**Participants:**

SLE patients with flares (n = 142) or bacterial infections (n = 106) were recruited in this retrospective study.

**Outcome:**

The peripheral blood of these patients was collected by the experimenter to measure the levels of routine examination indicators, immune cells, and cytokines. PLS-DA/OPLS-DA models and a bioscore system were established.

**Results:**

Both PLS-DA (R2Y = 0.953, Q2 = 0.931) and OPLS-DA (R2Y = 0.953, Q2 = 0.942) models could clearly identify bacterial infections in SLE. The white blood cell (WBC), neutrophile granulocyte (NEUT), erythrocyte sedimentation rate (ESR), C-reactive protein (CRP), procalcitonin (PCT), interleukin-6 (IL-6), IL-10, interferon-γ (IFN-γ), and tumor necrosis factor α (TNF-α) levels were significantly higher in bacteria-infected patients, while regulatory T (Treg) cells obviously decreased. A multivariate analysis using the above 10 dichotomized indicators, based on the cut-off value of their respective ROC curve, was established to screen out the independent predictors and calculate their weights to build a bioscore system, which exhibited a strong diagnosis ability (AUC = 0.842, 95% CI 0.794–0.891). The bioscore system showed that 0 and 100% of SLE patients with scores of 0 and 8–10, respectively, were infected with bacteria. The higher the score, the greater the likelihood of bacterial infections in SLE.

**Conclusions:**

The PLS-DA/OPLS-DA models, including the above biomarkers, showed a strong predictive ability for bacterial infections in SLE. Combining WBC, NEUT, CRP, PCT, IL-6, and IFN-γ in a bioscore system may result in faster prediction of bacterial infections in SLE and may guide toward a more appropriate, timely treatment for SLE.

## Introduction

Systemic lupus erythematosus (SLE) is a chronic autoimmune and multisystemic inflammatory disorder, which is characterized by multiple organ involvement and autoantibody secretion. Although both external factors, such as estrogenic hormones, pollution, and microorganisms, and internal factors, such as genes, have been investigated in the pathogenesis of SLE, the etiology of SLE remains to be elucidated (Illescas-Montes et al., 2019). The unbalanced autoimmune background and predominant immunosuppressive therapy give rise to susceptibility for infections in SLE (Wang et al., 2019). Approximately half of SLE patients develop infections during their disease progression, and bacterial infections remain the leading cause of morbidity and mortality in SLE (Allen et al., 2002; Furst et al., 2007; Feng et al., 2019). Distinguishing flares from bacterial infections in SLE remains a common dilemma for health care workers, in view of the clinical signs and symptoms of bacterial infections, such as fever, joint pain, and shortness of breath, mimicking those of flares in SLE. The empirical use of broad-spectrum antibiotics results in antibiotic resistance (Soni et al., 2013). Owing to the therapeutic regimens for bacterial infections being different from those for flares in SLE, timely diagnosis and correct treatment are crucial, and an improper treatment strategy may be fatal (Echeverri et al., 2018). Moreover, infections may contribute to the onset and exacerbations of SLE (Esposito et al., 2014).

While several biomarkers have been well demonstrated to differentiate bacterial infections and lupus flares in SLE (Hussein et al., 2010; Yang et al., 2010; Serio et al., 2014; Ajmani et al., 2019), there is a paucity of studies establishing integrated models or bioscore systems to effectively differentiate the two populations. Microorganism isolation serves as the confirmatory process for the detection of bacterial infections but has a long turn-around time and is susceptible to many factors, resulting in low sensitivity (Yang et al., 2016; Ye et al., 2016). Routine blood tests and serological indicators, such as white blood cells (WBC), the erythrocyte sedimentation rate (ESR), C-reactive protein (CRP), procalcitonin (PCT), interleukin-6 (IL-6), interleukin-10 (IL-10), and tumor necrosis factor α (TNF-α), have been assessed to diagnose bacterial infections in a short period (Marshall et al., 2003; Ospina et al., 2017; Thamphiwatana et al., 2017). A review by Baicus C concluded that the ratio of ESR/CRP >15 suggests lupus flares, while a ratio of less than 2 may indicate the occurrence of bacterial infections (Dima et al., 2016). However, no single biomarker has exhibited adequate sensitivity and specificity to serve as a standard tool for identifying bacterial infections (Holmes et al., 2003; Pierrakos and Vincent, 2010; Faix, 2011). Hence, the complexity of effectively distinguishing bacterial infections from lupus flares in SLE presents the need for an integrated method combining multiple biomarkers, or a more appropriate scoring system that can provide better insights into the diagnosis of bacterial infections in SLE.

In this study, we developed a novel, simple, and relatively accurate method to observe its capacity to distinguish bacterial infections from flares in SLE, by simultaneously integrating routine examination biomarkers, immune cell subpopulations, and serum cytokines, which were reported to be efficient in discovering the truth of bacterial infections.

## Methods

### Study Participants

In this retrospective study, 248 SLE patients were recruited, of which 221 were females and 27 males, with a median age of 38.00 years. They were diagnosed and admitted to the Second Hospital of Shanxi Medical University from January 2018 to December 2019, according to the American College of Rheumatology 1997 revised classification criteria for SLE. The disease activity of patients was evaluated based on Systemic Lupus Erythematosus Disease Activity Index (SLEDAI) (Bombardier et al., 1992), and a flare was regarded as three points higher than their previous SLEDAI (Bador et al., 2012). Out of the 248 SLE patients, 106 were thought to be infected by bacteria, and the criteria for bacterial infections were: 1) positive bacterial isolation in suspected infection sites; 2) typical clinical symptoms and signs of bacterial infections, such as fever, cough, sore throat, expectoration, diarrhea, pus discharge, dysuria, frequent and urgent urination; 3) imaging-positive results, including ultrasound, X-ray, and computed tomography; and 4) positive feedback on antibacterial treatment. Patients who were younger than 18 years of age, treated with antibiotics prior to admission, pregnant, or suffering from viral and fungal infections, cancer, or other autoimmune diseases were excluded. The ethics committee of the Second Hospital of Shanxi Medical University has approved our study (2016KY007), and all patients signed the informed consent.

### Routine Examination Biomarker

Peripheral blood samples were collected immediately after admission and before drug administration. Blood routine examination, including WBC, red blood cells (RBC), hemoglobin (Hb), platelet (PLT), lymphocyte (LYMP), and neutrophile granulocyte (NEUT), was performed using the Sysmex XN-9000 automated hematology analyzer. Complements 3 and 4 (C3, C4) and CRP were evaluated using the Beckman Coulter IMMAGE800 automatic protein analyzer. The levels of PCT and immunoglobulin (IgM, IgA, and IgG) were measured using the Beckman Coulter AU680 biochemical analyzer and ELISA, respectively.

### Immune Cell Subpopulations

For analyzing peripheral immune cell subpopulations, including CD3+T, CD4+T, CD8+T, B, and NK cells, we first added 50 μl EDTA-anticoagulated blood into Trucount tubes A and B (Becton-Dickinson, USA) respectively, whose own beads could eliminate the variation caused by manually adding reference beads before. CD3+T, CD4+T, and CD8+T cells were labeled with anti-CD3/CD4/CD8/CD45 antibodies in tube A. B and NK cells were identified by anti-CD3/CD16/CD56/CD45/CD19 antibodies in tube B. We collected these cells using flow cytometry (BD FACSCanto II) and analyzed them using the FACSCanto software. For Th1, Th2, and Th17 cells in the CD4+T subpopulation, we first used Ionomycin, PMA, and GolgiStop to stimulate the cells. Th1, Th2, and Th17 cells were then labeled by anti-CD4 and anti-IFN-γ/IL-4/IL-17 antibodies, respectively. Treg cells were identified with anti-CD4/CD25/FoxP3 antibodies. Finally, cells were collected using flow cytometry (BD FACSCalibur) and analyzed using the MultiSET software. Number of CD4+T subpopulations = the percentage of CD4+T subpopulations * the number of CD4+T cells.

### Cytokines

The concentrations of serum IL-2, IL-4, IL-6, IL-10, IL-17, TNF-α, and IFN-γ were detected using magnetic bead-based multiplex assays (Human Th1/Th2/Th17 subpopulation test kit: JIANGXI CELLGENE BIOTECH CO., LTD) following the manufacturer’s instructions. In brief, 25 μl of fluorescence detection reagent was incubated with an equivalent amount of EDTA anticoagulant plasma for 2.5 h at room temperature. We then used 1 ml of phosphate-buffered saline (PBS) to wash the mixture, and discarded the supernatant. The BD FACSDiva software was used to measure the levels of cytokines, and experimental data were analyzed using the FCAP software.

### Statistical Analysis

All data were analyzed using the SPSS22.0, SIMCA 14.1, and MedCalc softwares. Data were recorded as mean ± SD or median (Q_25_, Q_75_) for continuous variables, which were compared using the independent samples t-test or the Mann-Whitney U test. The Chi-square test was used to compare the difference between dichotomous variables. Partial least square discriminant analysis (PLS-DA) and supervised orthogonal PLS-DA (OPLS-DA) were conducted using the SIMCA 14.1 software to perform multivariate analysis of the data and further group the SLE patients with bacterial infections and lupus flares. Biomarkers containing more than 50% losing values were excluded. We normalized the data by mean centering and variance scaling. The seven-fold cross-validation (CV) approach was used to validate the multivariate models, and the R2Y, i.e., the explained ability of the model and Q2, i.e., the predictive power of the model, were calculated. Receiver operating characteristic (ROC) curves were drawn for the calculated significant indicators, to investigate their identification ability for bacterial infections in SLE. The cut-off value was selected by determining the point on the ROC curve closest to the ideal test of 100% sensitivity and specificity. The area under the ROC curve (AUC), sensitivity, specificity, positive likelihood ratio (PLR), negative likelihood ratio (NLR), positive predictive value (PPV), negative predictive value (NPV), and comparisons of these AUC were performed by MedCalc software. Significant indicators, based on the cut-off value of their respective ROC curve, were dichotomized to complete a multivariate analysis, which could screen out independent predictors and give different weights. Radar charts of the six independent predictors were drawn to characterize the bioscore distribution of SLE patients with bacterial infections and with lupus flares, visually demonstrating the advantage of the bioscore system in identifying bacterial infections in SLE. A two-sided *P*-value <0.05 was considered statistically significant.

## Results

### Demographic Data

**Figure 1:**
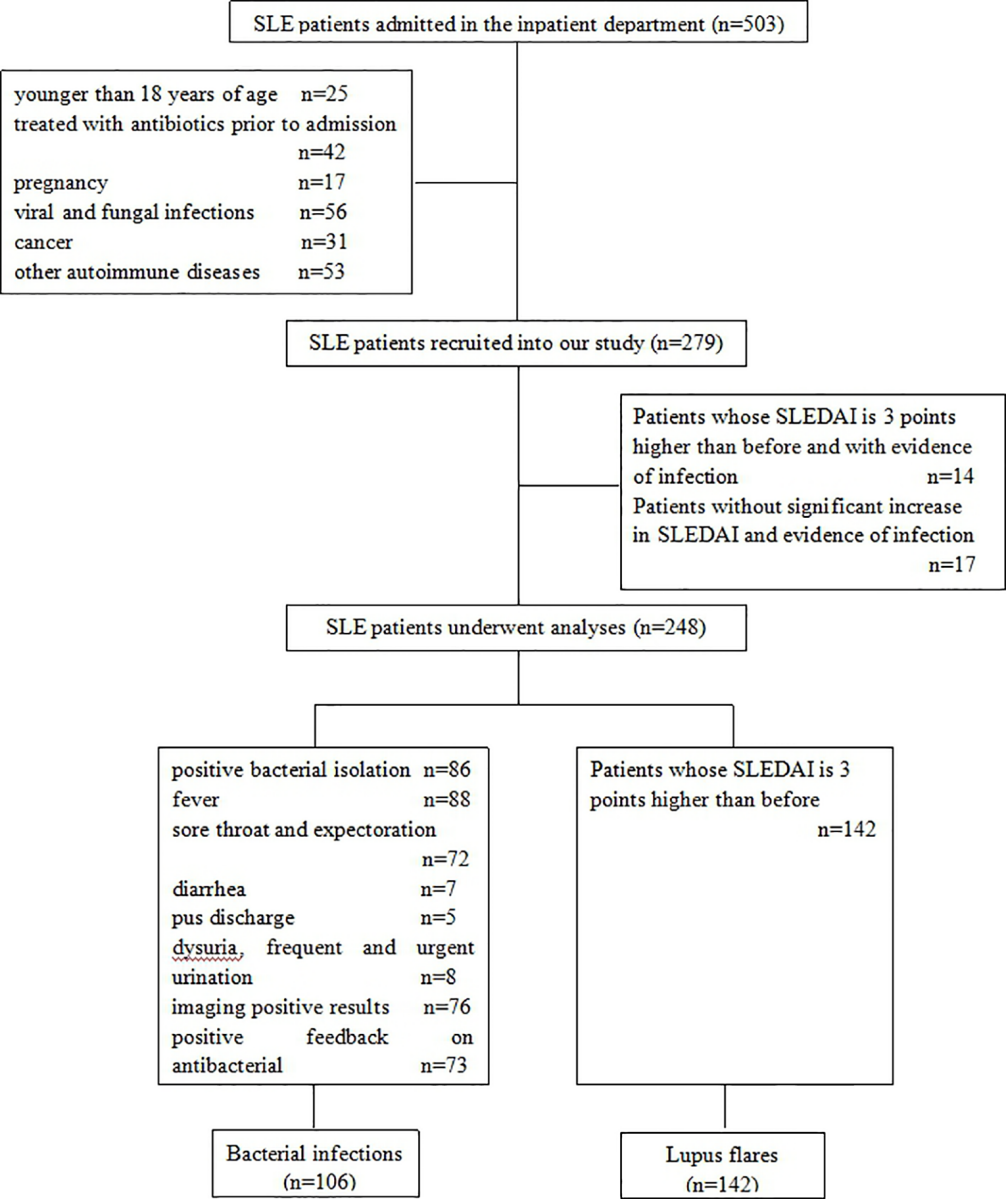
Flowchart for the inclusion and exclusion of SLE patients in the study.

Between January 2018 and December 2019, a total of 503 SLE patients were diagnosed and admitted to the Second Hospital of Shanxi Medical University. **Figure 1** showed the inclusion and exclusion criteria for SLE patients in the study. In total, 248 SLE patients were recruited in the study. Out of these, 142 (124 females and 18 males with a median age of 36.50 years) had lupus flares and 106 (97 females and 9 males with a median age of 39.00 years) were infected with bacteria, of which 78 presented respiratory infections, 8 presented urinary tract infections, 7 presented digestive tract infections, 5 presented soft tissue infections, and 8 presented bacteremia. No significant difference was found in age (*P* = 0.291) and sex distribution (*P* = 0.295) between the two groups. The demographic data and experimental results of patients were summarized in **Table 1**.

**Table 1:** Clinical characteristics and routine examination results of SLE patients.

	All (n = 248)	Bacterial Infections (n = 106)	Lupus Flares (n = 142)	*P*
**Clinical characteristics**				
Age	38.00 (29.00,50.00)	39.00 (31.00,51.00)	36.50 (28.00,50.00)	0.291
Gender: female/male	221/27	97/9	124/18	0.295
Hypertension (%)	88 (35.48%)	42 (39.62%)	46 (32.39%)	0.239
diabetes (%)	16 (6.45%)	9 (8.49%)	7 (4.93%)	0.259
Lupus nephritis (%)	146 (58.87%)	75 (70.75%)	71 (50.00%)	0.221
Cardiovascular disease (%)	74 (29.84%)	31 (29.25%)	43 (30.28%)	0.860
**Site of infection**				
Respiratory infection (%)		78 (73.58%)		
Urinary tract infection (%)		8 (7.55%)		
Digestive tract infection (%)		7 (6.60%)		
Soft tissue infection (%)		5 (4.72%)		
bacteremia (%)		8 (7.55%)		
**Routine examination**				
WBC (×10^9^/L)	4.69 (3.20,7.15)	5.46 (3.43,8.27)	4.45 (3.12,6.41)	0.006
RBC (×10^12^/L)	3.89 (3.41,4.29)	3.87 (3.26,4.26)	3.92 (3.45,4.37)	0.083
Hb (g/L)	112.95 ± 20.71	110.27 ± 20.42	114.95 ± 20.77	0.078
PLT (×10^9^/L)	174.50 (119.25,232.75)	166.50 (104.38,218.25)	179.50 (125.50,242.00)	0.222
LYMP (×10^9^/L)	0.99 (0.65,1.56)	0.90 (0.58,1.65)	1.02 (0.72,1.55)	0.316
NEUT (×10^9^/L)	2.95 (1.96,4.93)	3.55 (1.99,6.60)	2.78 (1,91,4.34)	0.026
C3 (g/L)	0.48 (0.33,0.68)	0.50 (0.30,0.70)	0.48 (0.33,0.67)	0.886
C4 (g/L)	0.10 (0.04,0.15)	0.11 (0.05,0.15)	0.10 (0.04,0.16)	0.771
IgG (g/L)	12.20 (9.31,17.40)	12.60 (9.80,19.40)	11.95 (9.21,16.18)	0.523
IgA (g/L)	2.49 (1.83,3.49)	2.77 (2.06,3.77)	2.40 (1.69,3.33)	0.089
IgM (g/L)	0.75 (0.52,1.22)	0.77 (0.51,1.33)	0.72 (0.52,1.10)	0.258
ALT (U/L)	18.70 (12.05,28.10)	19.85 (12.85,28.65)	17.60 (11.70,28.10)	0.386
AST (U/L)	22.55 (17.83,31.00)	24.15 (18.15,34.33)	21.60 (17.50,27.35)	0.112
ESR (mm/h)	40.00 (20.00,78.75)	50.00 (27.00,89.75)	30.50 (15.00,64.00)	<0.001
CRP (mg/L)	3.52 (2.17,9.18)	5.53 (2.74,19.47)	3.16 (2.00,6.45)	<0.001
PCT (ng/ml)	0.31 (0.18,0.49)	0.44 (0.25,0.71)	0.26 (0.16,0.37)	<0.001
**Disease activity**				
SLEDAI	11.00 (6.00,16.00)	11.00 (7.00,16.00)	12.00 (6.00,17.00)	0.924

Continuous data were compared by the Mann-Whitney U test and categorical data were analyzed by chi-square test.

WBC, white blood cell; RBC, red blood cell; Hb, hemoglobin; PLT, platelet; LYMP, lymphocyte cell; NEUT, neutrophile granulocyte; C3, complement C3; C4, complement C4; IgG, immunoglobulin G; IgA, immunoglobulin A; IgM, immunoglobulin M; ALT, alanine transaminase; AST, aspartic transaminase; ESR, erythrocyte sedimentation rate; CRP: C-reactive protein; PCT: procalcitonin; SLEDAI, SLE disease activity index.

### Predictive Models for Distinguishing Bacterial Infections From Lupus Flares in SLE

**Figure 2:**
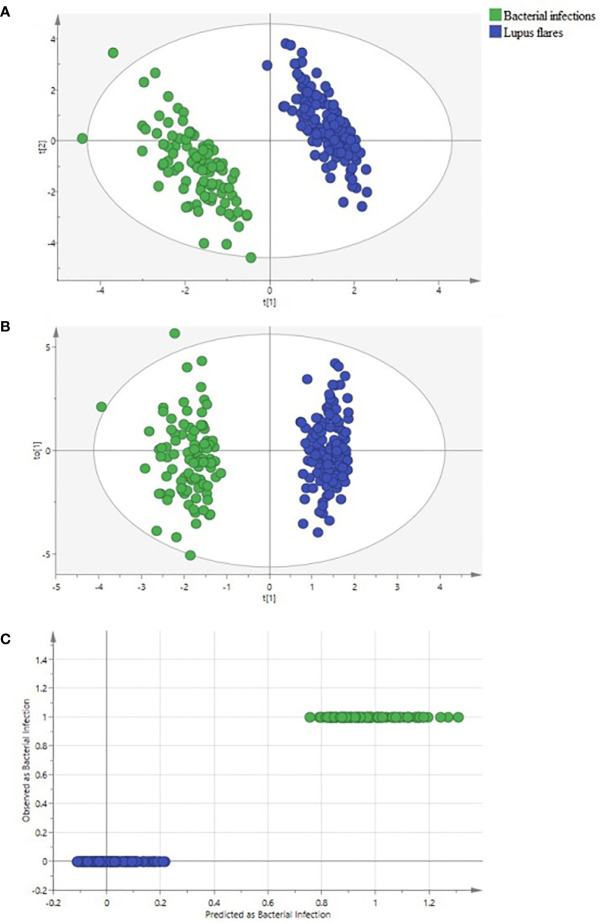
Identification of systemic lupus erythematosus (SLE) patients with bacterial infections and with lupus flares using partial least square discriminant analysis (PLS-DA) and orthogonal PLS-DA (OPLS-DA) methods. **(A)** the PLS-DA score scatter plot, **(B)** the OPLS-DA score scatter plot, and **(C)** the “Predicted *vs.* Observed” plot for distinguishing bacteria-infected SLE patients (green dots) from lupus flares (blue dots) based on 50 indicators. Both groups were greatly separated in the PLS-DA and OPLS-DA models. None of the SLE patients with lupus flares were predicted as a bacteria-infected SLE patient.

Both PLS-DA and OPLS-DA models, including the whole indicators to distinguish bacterial infections from lupus flares in SLE, were constructed. The following results were obtained in the PLS-DA model: R2Y = 0.996 and Q2 = 0.989 when the four major components were extracted; R2Y = 0.978 and Q2 = 0.963 when the three major components were extracted; and R2Y = 0.953 and Q2 = 0.931 when two major components were extracted, and each major component had a *P*-value below 0.05. In the OPLS-DA model, following results were obtained: R2Y = 0.996 and Q2 = 0.992 when the four major components were extracted; R2Y = 0.978 and Q2 = 0.976 when the three major components were extracted; and R2Y = 0.953 and Q2 = 0.942 when two major components were extracted, and the *P*-value of each component was less than 0.05. Taken together, we believe that the two models have sufficient explanatory ability and predictive power when two principal components are extracted from the two models (**Figures 2A, B**). Furthermore, the observed *vs.* predicted plot (**Figure 2C**) of the OPLS-DA model demonstrated that all SLE patients were correctly identified as having bacterial infections or flares, indicating that the model had a strong predictive ability for bacterial infections. Variable Important for the Projection (VIP) in the PLS-DA/OPLS-DA model is calculated to measure the influence intensity and explanatory ability of each indicator on the classification and discrimination of different groups, to assist the screening of meaningful variables. In general, a VIP value >1 may indicate a potential meaningful indicator. Based on the VIP values of the two models, we selected several indicators, as shown in **Tables 2**, **3**.

**Table 2:** Several indicators associated with bacterial infections in SLE based on VIP values in PLS-DA.

Indicators	VIP	Coefficient
CRP	2.812	0.100
ESR	2.283	0.082
WBC	1.959	0.070
IL-10	1.958	0.070
IL-6	1.958	0.065
NEUT	1.715	0.061
PCT	1.665	0.059
IFN-γ	1.663	0.059
TNF-α	1.231	0.044

VIP, Variable Important for the Projection.

**Table 3:** Several indicators associated with bacterial infections in SLE based on VIP values in OPLS-DA.

Indicators	VIP	Coefficient
CRP	2.380	0.070
IL-10	2.286	0.059
IFN-γ	2.172	0.044
TNF-α	1.816	0.035
WBC	1.799	0.082
ESR	1.618	0.065
IL-6	1.603	0.041
NEUT	1.257	0.059
PCT	1.284	0.100
Treg	1.125	-0.026

VIP, Variable Important for the Projection.

### Comparison of Various Indicators Between Bacterial Infections and Lupus Flares

**Figure 3:**
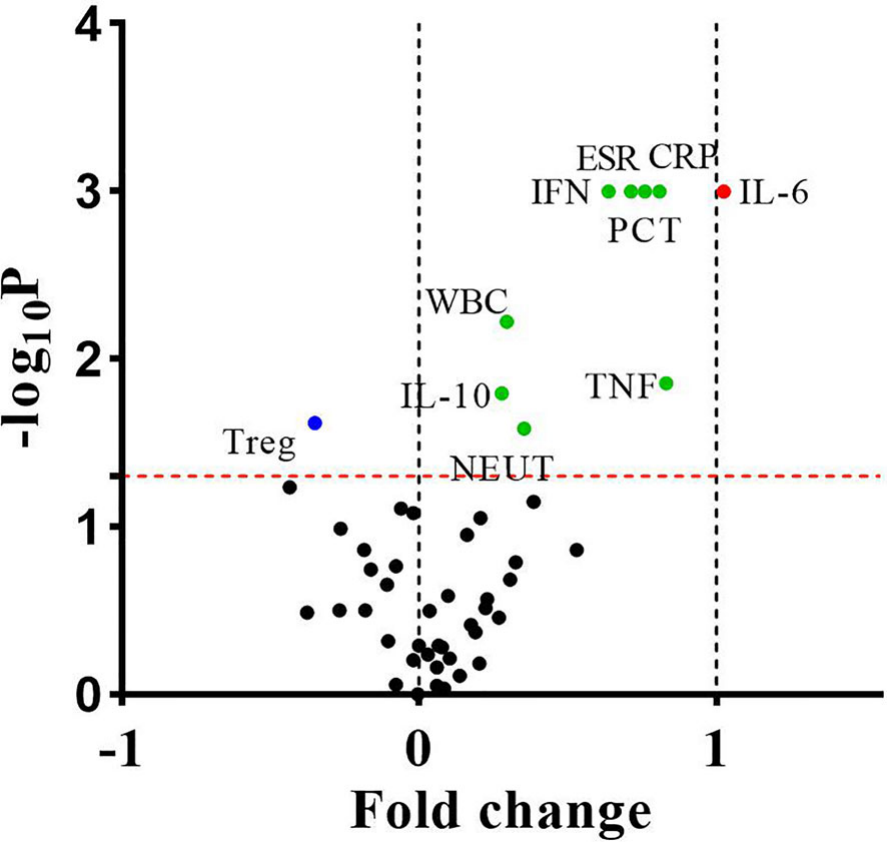
The differences of fifty indicators between 106 bacteria-infected patients and 142 lupus flares patients were showed in the volcano plot. The dots above the horizontal red dotted line indicated that these indicators were significantly different between the two groups (P < 0.05). The dots whose fold change <0 and >0 indicated that these indicators decreased or increased in infected patients, respectively, and the points of fold change >1 represented these indicators of infected patients increased by more than 2 times. *P* values were calculated by the independent samples t-test or the Mann-Whitney U test.

For routine examination indicators, the levels of WBC, NEUT, ESR, CRP, and PCT in the bacteria-infected group were significantly elevated compared to those in the lupus flares group (**Table 1**). For immune cells subgroups, the absolute number of Treg cells was lower in patients with bacterial infections obviously. For cytokines, the concentrations of IL-6, IL-10, IFN-γ, and TNF-α in the bacteria-infected group were significantly higher than these indicators in the flares group, respectively (**Figure 3**, **Table 4**).

**Table 4:** Characteristics of lymphocyte subpopulations and cytokine in SLE.

	All (n = 248)	Bacterial Infections (n = 106)	Lupus Flares (n = 142)	*P*
**Lymphocyte cells**				
T (cell/µl)	810.10 (523.79,1214.21)	703.45 (429.57,1238.44)	844.25 (606.83,1162.97)	0.103
B (cell/µl)	125.40 (66.90,232.00)	125.07 (57.47,231.19)	126.69 (68.37,234.85)	0.625
NK (cell/µl)	74.42 (43.81,118.06)	75.28 (42.64,116.70)	73.72 (44.32,119.92)	0.577
CD4^+^T (cell/µl)	359.13 (225.04,544.56)	293.01 (190.79,502.48)	395.98 (258.88,556.17)	0.058
CD8^+^T (cell/µl)	404.04 (247.07,608.09)	350.90 (208.54,609.10)	422.40 (277.90,609.88)	0.316
CD4^+^/CD8^+^ T cells	0.89 (0.60,1.26)	0.84 (0.56,1.18)	0.94 (0.61,1.37)	0.180
TBNK (cell/µl)	1,052.09 (713.44,1572.35)	979.56 (627.38,1540.93)	1,113.97 (813.16,1600.45)	0.137
T cells (%)	75.31 (67.27,82.41)	75.29 (65.59,82.88)	75.36 (68.59,82.36)	0.509
B cells (%)	13.55 (6.90,20.35)	14.51 (7.02,20.67)	12.73 (6.78,19.69)	0.424
NK cells (%)	7.38 (4.25,11.99)	7.53 (4.43,13.34)	7.20 (4.12,11.18)	0.509
CD4^+^T cells (%)	32.94 (25.33,39.00)	31.75 (24.45,37.95)	33.49 (26.46,39.86)	0.172
CD8^+^T cells (%)	37.15 (29.22,46.71)	38.64 (29.27,46.67)	36.00 (29.08,46.87)	0.608
TBNK (%)	98.20 (97.27,98.90)	98.00 (97.40,99.02)	98.27 (97.12,98.81)	0.997
Th1 (cell/µl)	55.80 (25.00,109.57)	59.17 (23.30,115.65)	55.80 (27.38,103.29)	0.922
Th2 (cell/µl)	2.79 (1.61,4.70)	2.66 (1.52,4.67)	2.86 (1.64,4.71)	0.479
Th17 (cell/µl)	4.35 (2.06,8.07)	3.73 (1.63,7.37)	4.84 (2.18,8.16)	0.325
Treg (cell/µl)	11.81 (7.05,18.54)	10.53 (6.58,16.52)	13.42 (7.95,19.94)	0.024
Th1 cells (%)	15.32 (9.23,24.61)	16.52 (8.95,30.21)	14.09 (9.29,23.51)	0.270
Th2 cells (%)	0.84 (0.62,1.05)	0.85 (0.67,1.09)	0.83 (0.60,1.03)	0.317
Th17 cells (%)	1.19 (0.64,2.06)	1.22 (0.64,2.14)	1.17 (0.64,2.00)	0.688
Treg cells (%)	3.27 (2.32,4.69)	3.42 (2.29,4.60)	3.26 (2.39,4.77)	0.928
Th1/Th2	19.89 (11.08,33.50)	21.95 (10.98,35.67)	19.08 (11.13,33.25)	0.651
Th17/Treg	0.37 (0.18,0.63)	0.36 (0.18,0.63)	0.38 (0.16,0.65)	0.871
Th1/Treg	4.63 (2.32,8.71)	5.00 (2.32,10.76)	4.15 (2.29,7.94)	0.348
Th2/Treg	0.26 (0.16,0.36)	0.28 (0.17,0.36)	0.24 (0.16,0.34)	0.306
B/Treg	10.32 (5.09,18.44)	11.48 (5.26,18.73)	9.29 (4.64,17.58)	0.207
NK/Treg	5.55 (2.72,11.17)	6.11 (3.21,12.78)	5.37 (2.55,10.85)	0.419
**Cytokine**				
IL-2 (pg/ml)	2.75 (1.66,5.21)	3.42 (1.86,5.62)	2.62 (1.49,4.99)	0.071
IL-4 (pg/ml)	2.70 (1.18,6.38)	3.27 (1.39,6.33)	2.61 (0.88,6.41)	0.163
IL-6 (pg/ml)	10.84 (6.12,22.23)	17.51 (8.67,35.97)	8.61 (5.20,15.01)	<0.001
IL-10 (pg/ml)	8.10 (4.92,12.54)	8.84 (5.16,15.33)	7.29 (4.67,11.85)	0.016
IL-17 (pg/ml)	7.68 (1.99,16.50)	9.02 (2.57,20.27)	6.25 (1.71,15.53)	0.138
IFN-γ (pg/ml)	5.53 (2.83,10.68)	6.97 (3.83,13.57)	4.48 (2.32,9.39)	0.001
TNF-α (pg/ml)	3.07 (1.33,7.41)	4.36 (1.68,8.62)	2.45 (1.09,6.56)	0.014

Continuous data were analyzed by the independent samples t-test or the Mann-Whitney U test, according to whether they satisfy normal distribution.

NK cells, natural killer cells; IL-2, interleukin-2; IL-4, interleukin-4; IL-6, interleukin-6; IL-10, interleukin-10; IL-17, interleukin-17; IFN-γ, interferon-γ; TNF-α, tumor necrosis factor-α.

### ROC Curves of the 10 Statistically Different Indicators

In total, there were 10 discriminative indicators that were screened out, based on PLS-DA/OPLS-DA models and univariate analysis. ROC curves of the above 10 statistically different indicators were plotted to explore their diagnostic efficiency for bacterial infections. The diagnostic efficiency of the 10 indicators, based on their cut-off values, were summarized in **Table 5**. The cut-off value for NEUT was set at 6.33 × 10^9^/L, with the highest specificity rate, 93.66%. IFN-γ, whose cut-off value was established as 4.50 pg/ml, had the highest sensitivity rate, 72.64%. PCT, whose cut-off value was set at 0.38 ng/ml, had the largest AUC (0.717).

**Table 5:** Variables of the ROC curve for multiple biomarkers.

biomarker	Cut-off value	AUC (%) 95% CI	*P*	Sensitivity (%) 95% CI	Specificity (%) 95% CI	PLR/NLR (%)	PPV/NPV (%)
WBC	>6.81 × 10^9^/L	0.602 (0.539–0.664)	0.006	39.62 (30.30–49.60)	80.99 (73.60–87.10)	2.08/0.75	60.90/64.20
NEUT	>6.33 × 10^9^/L	0.583 (0.519–0.645)	0.029	26.42 (18.30–35.90)	93.66 (88.30–97.10)	4.17/0.79	75.70/63.00
ESR	>35.00 mm/h	0.654 (0.592–0.713)	<0.001	69.81 (60.10–78.30)	55.63 (47.10–64.00)	1.57/0.54	54.00/71.20
CRP	>5.37 mg/L	0.645 (0.582–0.705)	<0.001	50.94 (41.00–60.80)	73.24 (65.20–80.30)	1.90/0.67	58.70/66.70
PCT	>0.38 ng/ml	0.717 (0.656–0.772)	<0.001	56.60 (46.60–66.20)	78.17 (70.50–84.70)	2.59/0.56	65.90/70.70
Treg	≤13.22 cells/µl	0.584 (0.520–0.646)	0.022	66.98 (57.20–75.80)	50.70 (42.20–59.20)	1.36/0.65	50.40/67.30
IL-6	>18.01 pg/ml	0.695 (0.633–0.751)	<0.001	49.06 (39.20–59.00)	85.92 (79.10–91.20)	3.48/0.59	72.20/69.30
IL-10	>12.34 pg/ml	0.590 (0.526–0.651)	0.015	33.96 (25.00–43.80)	81.69 (74.30–87.70)	1.85/0.81	58.10/62.40
IFN-γ	>4.50 pg/ml	0.619 (0.555–0.679)	0.001	72.64 (63.10–80.90)	50.70 (42.20–59.20)	1.47/0.54	52.40/71.30
TFN-α	>3.92 pg/ml	0.591 (0.527–0.653)	0.013	52.83 (42.90–62.60)	63.38 (54.90–71.30)	1.44/0.74	51.90/64.30

AUC, area under the ROC curve; CI, confidence interval; PLR, positive likely ratio; NLR, negative likely ratio; PPV, positive predictive value; NPV, negative predictive value; WBC, white blood cell; NEUT, neutrophile granulocyte; ESR, erythrocyte sedimentation rate; CRP, C-reactive protein; PCT, procalcitonin; IL-6, interleukin-6; IL-10, interleukin-10; IFN-γ, interferon-γ; TNF-α, tumor necrosis factor-α.

### Bioscore System for Identifying Bacterial Infections in SLE

**Figure 4:**
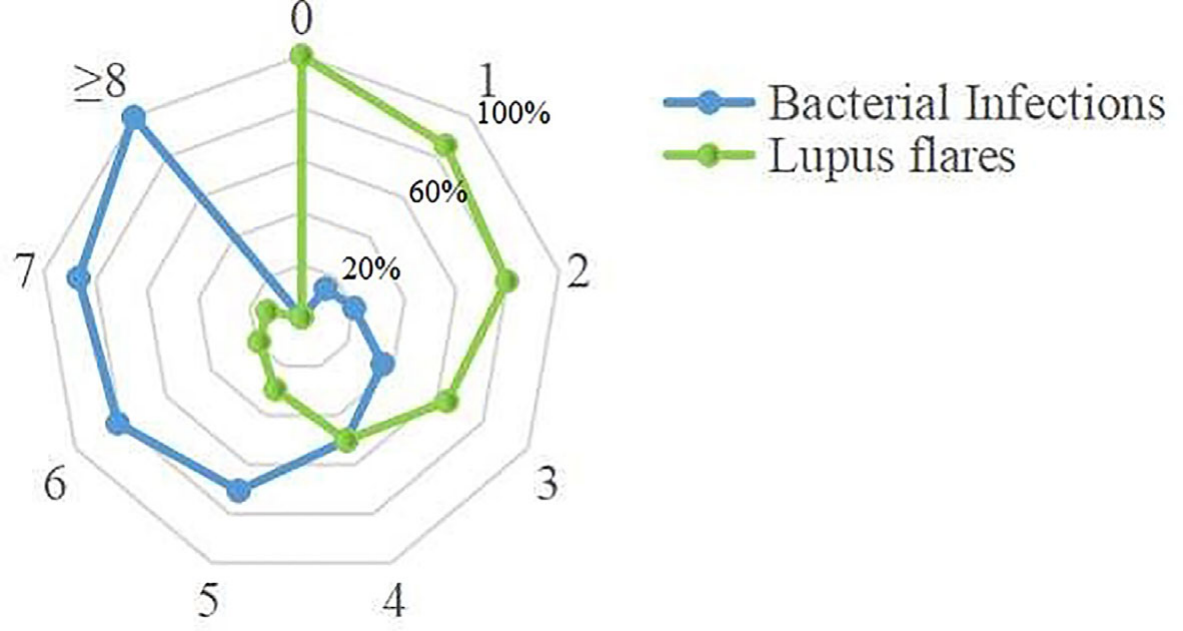
Distribution of the bacteria-infected SLE patients and the lupus flares patients in bioscore range 0~10. Twenty-seven patients with bioscore of 0 were lupus flares. All patients whose bioscore was greater than or equal to 8 (n = 13) were infected. A positive relationship was existed between the bioscore and the possibility of bacterial infections.

These 10 indicators, based on the cut-off value of their respective ROC curves, were dichotomized as high *versus* low. Each indicator, whose value was higher than its cut-off value, was classified as high and *vice versa*. Upon the inclusion of these 10 indicators in multivariate analysis, the results showed that WBC, NEUT, CRP, PCT, IL-6, and IFN-γ were independent predictors for bacterial infections in SLE (**Table 6**). Aiming to reveal the association, more concisely, between these indicators and the likelihood of bacterial infections in SLE, we created a simpler, faster, and more convenient bioscore system using the dichotomized versions of these six independent predictors. The indicator weight, which constitutes the bioscore system, was calculated by dividing the regression coefficient of each indicator by the minimum regression coefficient and rounded to the nearest rounded number. Each weight served as the score of each indicator (**Table 7**). The score of the bioscore system, hence, ranged from 0 to 10. The ROC curve of the bioscore system was plotted to observe the diagnostic ability for bacterial infections in SLE. The result revealed that the bioscore system could efficiently distinguish bacterial infections from lupus flares in SLE (AUC = 0.842, 95% CI 0.794–0.891). Furthermore, we scored the patients based on the weight of each indicator. The results showed that there were 0.00% and 100.00 of SLE patients with scores of 0, 8–10 infected with bacteria, respectively. There were 12 (85.71%), 47 (79.66%), and 27 (64.29%) patients with lupus flares among patients with bioscores of 1 (n = 14), 2 (n = 59), and 3 (n = 42), respectively. Both bacterial infections (n = 14) and lupus flares (n = 14) occurred in 50% of patients with a bioscore of 4 (n = 28). When the bioscore was >4, these patients were mainly infected with bacteria. The proportions of bacterial infections among SLE patients with scores 5, 6, and 7 were 70.59, 81.25, and 86.67%, respectively. The higher the bioscore, the greater the likelihood of bacterial infections in SLE (**Figure 4**).

**Table 6:** Multivariate analysis of the 10 dichotomized indicators.

	B	S.E.	Wald	df	*P*	Exp(B)	95.0% CI for EXP(B)	
							Lower	Upper
Variables in the Equation								
IL6	1.748	0.372	22.126	1	0.000	5.745	2.773	11.905
IFN-α	1.347	0.348	14.984	1	0.000	3.847	1.945	7.609
WBC	.971	0.422	5.283	1	0.022	2.641	1.154	6.043
NEUT	1.454	0.557	6.805	1	0.009	4.280	1.436	12.762
CRP	0.816	0.335	5.917	1	0.015	2.261	1.172	4.363
PCT	1.393	0.342	16.616	1	0.000	4.029	2.062	7.873
Variables not in the Equation								
IL-10				1	0.731			
TNF-γ				1	0.272			
ESR				1	0.190			
Treg				1	0.297			

EXP(B), odds ratio.

**Table 7:** Indicator Weights Used to build Bioscore.

Indicator	Score	Count (%)
WBC High Low	1 0	69 (27.82%) 179 (72.18%)
NEUT High Low	2 0	37 (14.92%) 211 (85.08%)
CRP High Low	1 0	92 (37.10%) 156 (62.90%)
PCT High Low	2 0	91 (36.69%) 157 (63.31%)
IL-6 High Low	2 0	72 (29.03%) 176 (70.97%)
IFN-γ High Low	2 0	147 (59.27%) 101 (40.73%)

## Discussion

The main results of our article included: 1) score plots of the PLS-DA and OPLS-DA models, including cytokines, lymphocyte subpopulations, and routine examination biomarkers, showed excellent performance in distinguishing between bacterial infections and lupus flares in SLE; 2) the levels of WBC, NEUT, ESR, CRP, PCT, IL-6, IL-10, IFN-γ, and TNF-α in bacteria-infected patients were significantly higher than in those with lupus flares, while the absolute number of Treg cells in bacteria-infected patients was obviously decreased; 3) the bioscore system constructed using the dichotomized versions of these 10 indicators provided a simpler, faster, and more convenient method to identify the SLE patients with bacterial infections.

Although the management of SLE patients has improved dramatically in recent years, bacterial infections remain the major cause of morbidity and mortality in SLE, given its own immune disorders and immunosuppressive intake. However, distinguishing between bacterial infections and lupus flares in SLE is still a common dilemma due to their similar symptoms and signs. Furthermore, in view of the disparate medication regimens, the potentially fatal consequences of unreasonable medication and the problem of antibiotic resistance caused by overuse and unnecessary use of antibiotics (Ospina et al., 2017), timely diagnosis, and discrimination of bacterial infections in SLE should be taken into consideration for developing specific therapies and achieving positive outcomes and cost effectiveness. Only a few studies have reported methods for effective distinction between bacterial infections and lupus flares in SLE in the past, and they have only explored the indicators, including WBC, CRP, PCT, and nCD64, independently (Hussein et al., 2010; Yang et al., 2010; Serio et al., 2014; Ajmani et al., 2019). Given the limitations of microbial culture and routine laboratory indicators, depending on a single biomarker to identify bacterial infections is not a reliable strategy. We have previously reported that combining multiple indicators to effectively distinguish bacterial infections from lupus recurrence; however, we did not take into account the role of each indicator in identifying bacterial infection (Feng et al., 2019). Therefore, seeking a more comprehensive data model to distinguish the bacterial infections in SLE is imperative.

Serving as indicators that are known to be elevated in bacterial infections, the levels of WBC and NEUT, in our study, were significantly higher in infected patients. However, the WBC count detected with the conventional method only reflects 5% of the whole WBC number, and a reduced WBC number in patients with bacterial infections has also been reported in several studies (Ye et al., 2016). ESR, which was significantly elevated in the infected group in the current study, is susceptible to a variety of factors. The change in ESR, consequently, could not specifically reflect the occurrence of bacterial infections. As an acute phase response protein, CRP has been reported to be elevated in the early phase of bacterial infections (Yang et al., 2016; van der Does et al., 2018), which was consistent with our findings. However, normal CRP levels were also observed in local and mild infections (Ng et al., 2000; Venkatesh et al., 2010). PCT, which rapidly increases as soon as bacterial infections commence, has also been reported to have no significant difference between SLE patients with bacterial infections and those with lupus flares (Lanoix et al., 2011). In recent years, there has been a gradual focus on the diagnostic ability of inflammatory cytokines for bacterial infections. Several Th1/Th2 cytokines have been found to be sensitive to the risk of bacterial infections (Santolaya et al., 2008; Tang et al., 2011; Urbonas et al., 2012a; Urbonas et al., 2012b). Indeed, the levels of IL-6, IL-10, IFN-γ, and TNF-α in our study, showed an upward trend. The changes in the levels of these cytokines were in line with previous reports. Additionally, Tang Y. et al. have also demonstrated that IL-6 and IL-10 could serve as important predictors for the infection severity and prognosis of bacteria-infected patients (Tang et al., 2011). Pro-inflammatory Th17 cells and suppressive Treg cells are involved in the immune response process (Zhou et al., 2008; Mubarak et al., 2016). The upregulation of Th17 or downregulation of Treg cells may trigger immunity to bacterial infections (Kimura and Kishimoto, 2010). Treg cells in bacterial-infected patients were significantly downregulated in our study, while no significant changes in Th17 cells were observed. We speculate that the reason for this behavior of Treg cells may be that decreased Treg cells are required to maintain the function of Th17 cells to clear bacteria during bacterial infections. Interestingly, Liu Y. et al. found that the numbers of Th17 and Treg cells in the infected group were significantly higher than those in the non-infected group (*P* < 0.001).

Previous studies have reported that the PLS-DA/OPLS-DA model was used to process multiple protein indicators to effectively predict bacterial infections (Kuusela et al., 2017; Tong et al., 2019). In our study, the PLS-DA/OPLS-DA models showed clear identification ability for bacterial infections in SLE. In addition to building the PLS-DA/OPLS-DA models, ROC curve analyses were also conducted to observe the identification ability of the 10 indicators for bacterial infections. For a single biomarker, NEUT had the highest specificity rate, 93.66%. IFN-γ exhibited a relatively higher sensitivity of 72.64%. However, the AUCs of these 10 indicators were not large enough to be strongly persuasive.

To enable clinicians to make rapid judgments for bacterial infections, we have constructed a bioscore system based on the different weights of these six independent predictors, which had the great predictive ability (AUC = 0.842). An advantage of this system is that the shortage of individual biomarker for identifying bacterial infections was overcome, and its prediction power is even more convincing. The bioscore system, in our study, showed a positive relationship between the bioscore and the possibility of bacterial infections, and a bioscore ≥8 can be used as a powerful indicator for the diagnosis of bacterial infections, which would be a powerful aid to physicians in handling patient decisions.

There were some deficiencies in our study. First, due to the retrospective nature of this study, a selection bias may have existed. Second, the clinical manifestation of patients, who were enrolled through the criteria “clinical improvement after antibiotic treatment,” may have also improved without antibiotic treatment. Therefore, the number of patients in the bacteria-infected group may have been overestimated. Third, the power of the bioscore system to identify new patients with bacterial infections has not been validated yet.

## Conclusion

The PLS-DA/OPLS-DA models, including the above biomarkers, showed a strong predictive ability for bacterial infections in SLE. Combining WBC, NEUT, CRP, PCT, IL-6, and IFN-γ in a bioscore system may result in a faster prediction of bacterial infections in SLE and may guide toward a more appropriate, timely treatment for SLE.

## Data Availability Statement

The original contributions presented in the study are included in the article/supplementary material. Further inquiries can be directed to the corresponding author.

## Ethics Statement

The studies involving human participants were reviewed and approved by the ethics committee of the Second Hospital of Shanxi Medical University. The patients/participants provided their written informed consent to participate in this study.

## Author Contributions

JL came up with the idea. XWZ and MF performed the detection of cytokines and the writing of the article. HG performed the writing of the article. ZL, YW, and YQ completed the detection of the immune cell subpopulations. YW and XCZ contributed to the analysis of data. CG polished the article. All authors contributed to the article and approved the submitted version.

## Funding

The study was supported by the Preferential Financed Projects of Shanxi Provincial Human Resources and Social Security Department (2016-97); the Scientific Research Project of Shanxi Health Planning Committee (201601042); the Scientific Research Foundation for the Returned Overseas Scholar of Shanxi Province (2017-116); Scientific Research Project of Shenzhen University General Hospital (0000040522); the Key Research and Development Project (Guide) of Shanxi Province (201803D421067); Shanxi Applied Basic Research Program Project (201901D111377); Scientific research project of Shanxi Provincial Health Commission (2019044), and Shanxi Province Research support project for returned Overseas Students (2020-191).

## Conflict of Interest

The authors declare that the research was conducted in the absence of any commercial or financial relationships that could be construed as a potential conflict of interest.
